# Antihyperglycemic Effects of Short Term Resveratrol Supplementation in Type 2 Diabetic Patients

**DOI:** 10.1155/2013/851267

**Published:** 2013-09-01

**Authors:** Ali Movahed, Iraj Nabipour, Xavier Lieben Louis, Sijo Joseph Thandapilly, Liping Yu, Mohammadreza Kalantarhormozi, Seyed Javad Rekabpour, Thomas Netticadan

**Affiliations:** ^1^Department of Endocrine and Metabolic Diseases, The Persian Gulf Tropical Medicine Research Center, Bushehr, University of Medical Sciences, Bushehr 7514763448, Iran; ^2^Heart Failure Research Laboratory, Canadian Centre for Agri-Food Research in Health and Medicine, St. Boniface Research Centre, Winnipeg, MB, Canada R2H 2A6; ^3^Agriculture and Agri-Food Canada, Winnipeg, MB, Canada R3T 2M9; ^4^Department of Physiology, University of Manitoba, Winnipeg, MB, Canada R3E 0J9

## Abstract

The objective of this study was to examine the effectiveness of resveratrol in lowering blood glucose in the presence of standard antidiabetic treatment in patients with type 2 diabetes, in a randomized placebo-controlled double-blinded parallel clinical trial. A total of 66 subjects with type 2 diabetes were enrolled in this study and randomly assigned to intervention group which was supplemented with resveratrol at a dose 1 g/day for 45 days and control group which received placebo tablets. Body weight, blood pressure, fasting blood glucose, haemoglobin A1c, insulin, homeostatic assessments for insulin resistance, triglycerides, total cholesterol, low density lipoprotein, high density lipoprotein, and markers of liver and kidney damage were measured at baseline and after 45 days of resveratrol or placebo supplementation. Resveratrol treatment significantly decreased systolic blood pressure, fasting blood glucose, haemoglobin A1c, insulin, and insulin resistance, while HDL was significantly increased, when compared to their baseline levels. On the other hand, the placebo group had slightly increased fasting glucose and LDL when compared to their baseline levels. Liver and kidney function markers were unchanged in the intervention group. Overall, this study showed that resveratrol supplementation exerted strong antidiabetic effects in patients with type 2 diabetes.

## 1. Background

The incidence of diabetes mellitus continues to rise worldwide, and it has far reaching consequences to the quality of human life [1, 2]. Progression of uncontrolled diabetes culminates in microvascular (retinopathy, nephropathy, and neuropathy) and macrovascular (cardiac, cerebral, and peripheral vascular disease) complications and premature death [3, 4]. Among the two forms of diabetes, type 2 diabetes mellitus (T2DM) (formerly called non-insulin-dependent diabetes mellitus) is the most prevalent one and is primarily characterized by insulin resistance and associated hyperglycemia [5–7].

The most recent epidemiological data from the International Diabetes Federation (IDF) has revealed that diabetes currently affects around 371 million people globally and 4.8 million die every year [8]. Moreover, it has been proposed that developing countries will contribute to the majority (77.6%) of the diabetic patients globally, by the year 2030 [9, 10]. Among these countries, Islamic Republic of Iran is one of the countries having the highest prevalence of T2DM [11, 12]. These alarming epidemiological figures demonstrate the need of alternative therapeutic strategies, dietary interventions, and life style modifications, in addition to the existing pharmacological agents to prevent/manage T2DM in these high risk populations.

In the past decade, resveratrol, a naturally occurring polyphenolic compound, found predominantly in grapes and peanuts, has been widely documented in the scientific literature for its beneficial effects in preventing and/or managing several diseases [13, 14]. Over the past 6 years, we have reported potent effects of resveratrol in preventing or reversing cardiac impairment in animal models of hypertension and obesity [5, 15, 16]. In the obese model [5], we also reported that resveratrol reduced hyperglycemia. Several other studies have also reported improvement in metabolic parameters in resveratrol treated animal models of insulin resistance including T2DM [17–20]. 

Despite the strong evidence for resveratrol generated from preclinical research and its widespread use as a nutritional supplement, well designed human clinical trials investigating the antidiabetic effects of resveratrol are limited. To date, only two human studies have been completed to investigate the potential of resveratrol in T2DM patients [21, 22]. Both studies [21, 22] reported a modest reduction in blood glucose levels, while one showed no effect on insulin levels [22]. Recently, Crandall et al. also showed a slight improvement in insulin sensitivity and postprandial plasma glucose in resveratrol treated prediabetic older adults [23]. However, two human clinical trials investigating the metabolic effects of resveratrol on insulin sensitivity and glucose response in obese subjects reported no clinically significant effect on blood glucose [24, 25]; it must be noted that these studies included relatively healthy (nondiabetic) subjects, and therefore the results cannot be extrapolated to make decisive conclusions regarding the antidiabetic potential of resveratrol. Given the discordant results achieved with resveratrol treatment, further human clinical trials are needed to establish the antidiabetic effects of resveratrol.

As stated earlier the increased risk and prevalence of T2DM among Iranians make them an ideal study population to test the efficacy of resveratrol in improving diabetes related metabolic abnormalities. Accordingly, the primary objective of the present study was to examine whether supplementation with resveratrol at a dose 1 g/day would have a clinically apparent blood glucose lowering effect, while the secondary objective was to assess the effects on insulin, metabolic markers, and cardiovascular risk factors. This study will also address whether resveratrol could complement the standard antidiabetic regimen [26] and render added protection in patients with T2DM, in a randomized placebo-controlled, double-blinded parallel clinical trial.

## 2. Methods

### 2.1. Subjects and Study Design

The study was approved by the Medical Ethics Committee of Bushehr University of Medical Sciences. The project was also registered with Clinical Trial Registry of Iran (registration no: IRCT201111198129N1). The informed consent was obtained from each patient at the time of enrolment and continued as a process throughout the investigation. Also, the patients had free medical care and consultation during the study period, especially in the case of any adverse reaction or complications.

Seventy patients with T2DM who repeatedly visited the Endocrine Clinic of the Persian Gulf Tropical Medicine Research Center, Bushehr University of Medical Sciences, Bushehr, Iran, were enrolled in a randomized placebo-controlled double-blinded single centre clinical trial. The following inclusion criteria were used to recruit the subjects who had T2DM: (1) age between 20 and 65 years, (2) minimum of 6 months on oral hypoglycemic treatment or combination therapy, (3) being not on any antioxidant therapy such as vitamin supplements, and (4) having no allergy to grapes, green tea, and peanuts. Patients with type 1 diabetes, pregnant women, lactating mothers, and patients with severe heart disease, hepatic disease, and renal impairment were excluded from the present study. The patients were randomly assigned to control and intervention groups. 

#### 2.1.1. Randomization/Blinding Procedures

Stratified randomization was used to allocate participants to the 2 treatment sequences so that equal numbers of men and women will be allocated to each sequence. Thereafter, randomization of the participants into treatment group and placebo group was done by computer generated numbered sequence codes which were given in opaque envelopes to subjects in the first study visit. Both the clinical team and participants were blinded from the time of randomization until analysis was completed.

#### 2.1.2. Compliance

At each visit (once a week during the intervention period), the participant returned unused capsule bottles. Study compliance was assessed by counting the remaining capsules from the bottle every week. Every participant was asked to complete a questionnaire during each visit to monitor the type of foods consumed during the study, particularly to confirm that they did not have food products that may contain resveratrol. No other assessments were done during these routine visits.

### 2.2. Treatment Regime

The patients in the intervention group received 500 mg, twice a day (a total of 1 g/day) of resveratrol capsules (99% pure, Biotivia, Bioceuticals International SrI, Italy) for a period of 45 days. The control group received 500 mg twice daily of placebo capsules (totally inert microcellulose, Biotivia, Bioceuticals International SrI, Italy) supplementation for the same period of time. Since the study was also intended to test the effectiveness of resveratrol when administered in conjunction with existing treatments against T2DM, all patients were allowed to continue their existing antidiabetic medications during the course of the study. Oral hypoglycemic agents and insulin were not modified during the course of the study. Among the participants who completed the study, in the control group, 12, 7, 10, and 2 patients were on metformin, glibenclamide, metformin + glibenclamide, and insulin + metformin, respectively. In the intervention group, 9, 10, 12, and 2 patients were on metformin, glibenclamide, metformin + glibenclamide, and insulin + metformin, respectively. Few subjects were also under atorvastatin (*n* = 3), atenolol (*n* = 1), simvastatin (*n* = 1), and lovastin (*n* = 1). 

### 2.3. Physical Measurements

Blood pressure was assessed twice on the right arm after a 15-minute rest in the sitting position, using a standard mercury sphygmomanometer. Height was measured using a stadiometer and weight using standard weighing balance. Heavy outer garments and shoes were removed before height and weight were measured. Body mass index (BMI) was calculated as weight in kilograms divided by the square of height in meters using Global Database on BMI (World Health Organization, 2006).

### 2.4. Biochemical Measurements

Fasting blood samples (12 hours) were taken at the baseline and after 45 days of treatment. All samples were promptly centrifuged, and plasma and serum were separated and kept frozen at −80°C until used. Analyses for biochemical parameters (blood glucose, triglyceride, and cholesterol levels) were carried out at the Persian Gulf Tropical Medicine Research Center on the day of blood collection using a Selectra 2 autoanalyzer (Vital Scientific, Spankeren, The Netherlands). Glucose levels were measured with the enzymatic (glucose oxidase) colorimetric method using a commercial kit (Pars Azmun Inc., Tehran, Iran) [27]. Creatinine levels were estimated using enzymatic method. Serum total cholesterol and high density lipoprotein (HDL) cholesterol were estimated using cholesterol oxidase phenol aminoantipyrine enzymatic method and triglycerides using the glycerol-3-phosphate oxidase phenol aminoantipyrine enzymatic method [27]. Serum low density lipoprotein (LDL) cholesterol was calculated using the Friedewald formula [28].

In order to measure liver function in the patients, the enzymes serum glutamic oxaloacetic transaminase (SGOT), serum glutamic pyruvic transaminase (SGPT), alkaline phosphatase (ALP), and gamma-glutamyltransferase (GGT) were measured by enzyme kinetic methods [27]. Intra- and interassay coefficients of variation (CVs) were 2.36%, 2.16%, 3.28%, 1.87%, 1.16%, 1.1%, 1.43%, and 0.9%, respectively. Hemoglobin A1c (HbA1c) in the whole blood sample of patients was measured using the boronate affinity assay kit (Nycocard HbA1c, Axis-SHIELD poc AS, Norway) [29]. Serum insulin levels were estimated using the electrochemiluminescence immunoassay “ECLIA” kit (Elecsys and Cobbas, Roche Diagnostics Gmbh, Sandhofer straße 116, Mannheim, Germany) [30]. The assay sensitivity was 1.76 *μ*IU/mL; the intra- and interassay coefficients of variance were 1.79–2.6% and 2.88–5.99%, respectively. Insulin resistance was assessed by calculating the homeostasis model of assessment index (HOMA-IR) using the following equation: fasting insulin (*μ*IU/mL) × fasting glucose (mg/dL)/405. Also, the percentage of beta-cell function from fasting serum glucose and insulin concentrations was assessed by calculating homeostasis model of assessment index (HOMA-*β*) using the following equation: 360 × fasting insulin (*μ*IU/mL)/fasting glucose (mg/dL) −63 [31]. 

### 2.5. Statistical Analysis

Normal distribution of the data was controlled with the Kolmogorov-Smirnov test. Probability values <5% were considered statistically significant. For categorical variables, the two groups were compared with contingency table analyses using chi-square statistics. For continuous variables, a two-tailed *t*-test was used to compare groups. The paired Student's *t*-test was used to compare the means of two variables for a single group. Since Friedewald formula was used to obtain the values for LDL-C levels and this formula was not applicable for the TG values more than 300 mg/dL, repeated measurement analysis was used only for the lipid profiles to have normal distribution and to check the comparison of the effect of drugs during the time of experiment and also to compare the effect between the two groups. Also, because the lipid profiles had normal distribution, standard deviation was preferred rather than interquartile ranges. The Mann-Whitney *U* Test was used to compare differences between two independent groups when the dependent variable was continuous but not normally distributed. The Wilcoxon signed-rank test was used as an alternative to the paired Student's *t*-test for dependent samples when the variables cannot be assumed to be normally distributed. All statistical analyses were performed using the PASW Statistics GradPack 18 (SPSS Inc., Chicago, IL, USA). All data is represented as means ± SD except for Table 4. In Table 4, the number of patients in each subgroup was less than enough to use parametric statistics. The Mann-Whitney *U* Test (nonparametric statistics) was used, and the results are reported as Interquartile to have normal distribution among the patients in different subgroups.

## 3. Results

**Figure 1:**
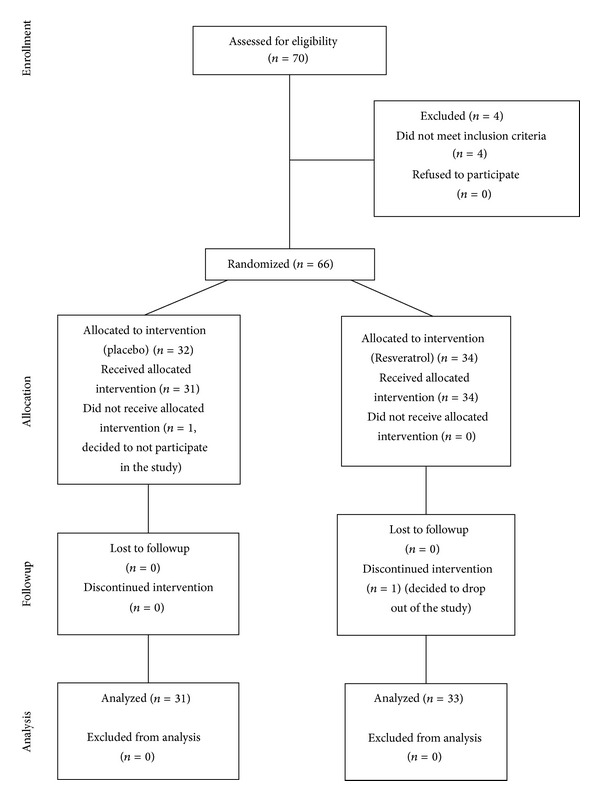
Consort diagram of the human trial.

There was one subject dropout from each group during the course of the study. At the end of the study, there were 31 and 33 subjects in control and intervention group, respectively. A consort diagram is presented showing the flow of participants through the study (Figure 1).  Table 1 shows the general characteristics of the intervention group compared with the controls. At the baseline level, there was no significant difference between the two groups regarding age, gender, bodyweight, BMI, systolic and diastolic blood pressure, duration of disease, smoking, history of hypertension, HbA1c, serum triglyceride, insulin, HOMA-*β*, creatinine, and HDL-C levels. However, when compared with controls, the intervention group had significantly higher fasting blood glucose, HOMA-IR, total cholesterol, and LDL-C levels. The prevalence of diabetes mellitus in the family was higher among the controls than the intervention group (*P* < 0.05, Tables 1 and 2). None of the participants had a history of alcohol consumption.

Resveratrol and placebo supplementations had no effect on body weight and BMI in the intervention and control groups, respectively (Table 2). Systolic blood pressure was significantly reduced with resveratrol supplementation, while the control groups had no change in systolic blood pressure, when compared to their respective baseline levels (Table 2). Diastolic blood pressure was unchanged in both groups throughout the study (Table 2). Liver function markers (SGOT, SGPT, GGT, and ALP) and a kidney function marker (creatinine) were unchanged with resveratrol treatment for 45 days in T2DM patients (when compared with baseline values). At the end of the study, ALP was significantly increased in the control group (Table 2). Glucose levels were significantly reduced with resveratrol treatment in T2DM patients (when compared with baseline levels); patients in control group showed a small but significant increase in glucose levels (Table 2). There was a significant decrease in HbA1c in intervention group when compared to the baseline levels, whereas, there was no change in HbA1c levels in control group (Table 2). Insulin levels in intervention group were significantly decreased when compared to baseline levels, while control group had no change in insulin levels (Table 2). Homeostatic model assessment of insulin resistance (HOMA-IR) and beta cell function (HOMA-*β*) showed that both parameters were significantly decreased in intervention group when compared to the baseline levels (Table 2); however, HOMA-IR did not change in control group, while, HOMA-*β* marginally but significantly decreased (Table 2).

When compared with baseline, HDL levels were significantly increased in the intervention group. Changes in total triglycerides, cholesterol, and LDL levels showed a decreasing trend but were not statistically significant (Table 2). However, the control group had increased LDL levels at the end of the study when compared to the baseline levels, while triglycerides, total cholesterol, and HDL were unchanged (Table 2).

A comparison of the changes in the anthropometric, clinical, and biochemical measurements during the study period (end of the study period minus baseline) between the intervention and control groups are shown is Table 3. When compared to the controls, a significant decline was observed in systolic blood pressure, HbA1c, HOMA-IR, serum fasting glucose, and insulin, while significant increment was observed in the levels of HDL-C (*P* < 0.0001) in the intervention group. When compared to the control group, no significant changes were found for BMI, body weight, diastolic blood pressure, HOMA-*β*, levels of total cholesterol, creatinine, and liver enzymes in the subjects supplemented with resveratrol. 

Subgroup analyses were also done to determine if resveratrol had any additive effect when combined with metformin and glibenclamide. The seven parameters that showed significant changes were evaluated in subgroup analyses. Subgroup analyses concluded that resveratrol had an additive effect on the metabolic parameters; however, this additive effect was independent of the type of hypoglycemic agents (Table 4). Only four patients were on insulin treatment, two in the intervention group and two in the control group; therefore, the insulin group was not included in the subgroup analyses.

## 4. Discussion

To date, numerous studies testing the effectiveness of resveratrol on cell and animal models have demonstrated the potency of resveratrol against different ailments [32]. Based on the outcomes of recent clinical trials, there is now a reasonable amount of scientific evidence to support the claim that resveratrol is beneficial against chronic diseases [33]. Among the human studies mentioned earlier in the introduction, Bhatt et al. [21] and Brasnyó et al. [22] reported that treatment with resveratrol 250 mg/day [21] and 10 mg/day [22], respectively, resulted in an improvement in metabolic parameters in T2DM patients. However, both the studies had few limitations; for example, Bhatt et al. lacked a placebo group, while Brasnyó et al. had only 19 subjects with only male subjects recruited. Another study done by Crandall et al. [23] showed some effects on insulin sensitivity and postmeal glucose levels in prediabetic older adults. Poulsen et al. [25] reported that resveratrol supplementation in obese but healthy males did not have any beneficial effects on metabolic markers while, Timmers et al. [24] did show some metabolic changes in resveratrol supplemented healthy obese men. Crandall et al. and Poulsen et al. [23, 25] used resveratrol at a dosage around 1.5 g/day, while Timmers et al. study used 150 mg/day dose for supplementation. Given the varied nature of results on the effectiveness of resveratrol as glucose lowering agent in obese/diabetic patients, it was important to establish if resveratrol indeed has antihyperglycemic effects in human subjects.

The results of the current study showed that resveratrol supplementation at a dose of 1 g/day had a remarkable effect in lowering glucose levels, along with major improvements in other metabolic parameters in humans with T2DM. Given the fact that there is no conclusive data on an effective dosage of resveratrol when used as a supplement, we used a dose that is moderately high (1 g/day) in order to give us a better chance to observe clinically apparent antidiabetic effects of resveratrol. General characteristics at the baseline level were not significantly different between the two groups, with the exceptions of fasting blood glucose, HOMA-IR, total cholesterol, and LDL-C levels, which were higher in intervention group. The control group had a higher prevalence of diabetes mellitus in the family, while none of the participants had a history of alcohol consumption. None of these parameters were expected to affect the final result.

Throughout the study, there were no significant changes in the physical characteristics of the subjects in both groups. The drop in systolic blood pressure in resveratrol treated type 2 diabetic patients is consistent with that observed by Bhatt et al. [21]. The small but significant reduction in blood pressure observed might be due to the improvement in insulin resistance and glucose homeostasis [34]. The effectiveness of resveratrol as a blood pressure lowering agent at the current dosage needs to be independently tested on hypertensive subjects.

As mentioned earlier in the discussion, one of the key findings of this study is a marked drop in glucose levels with resveratrol treatment in T2DM patients. Patients in control group had a small but significant increase in glucose levels compared to the baseline (please note that the dosage and the type of the medication were not modified during the study period). Taken together, these results strengthen the case for use of resveratrol as a supplement in conjunction with existing antidiabetic therapies to manage glucose levels in T2DM patients. In comparison to the earlier results [21–23], our data showed strong glycemic control with resveratrol supplementation. Accordingly, it may be suggested that short term supplementation with a moderate to high dose of resveratrol (1 g/day) as used in the current study may help to markedly lower blood glucose levels. We speculate that, once blood glucose levels are normalized, T2DM patients may be administered a lower dose (less than the 1 g/day) of resveratrol that in conjunction with dietary modifications and appropriate exercise regimen may help maintain lowered blood glucose levels. Whether the use of a lower dose is an effective option in the long term is a question that needs to be tested in an independent clinical trial.

A major concern in using higher doses of resveratrol in humans would be the possibility of toxic effects to major organs in the body. However, in a recent well-designed dose-response study, Brown et al. reported that up to 1 g resveratrol was well tolerated in human subjects and did not result in toxic effects, while 2.5 and 5 grams resulted in some gastrointestinal symptoms [35]. Brown et al. study was conducted on healthy subjects, while another study on older adults showed that 1-2 g/day dosage of resveratrol was well tolerated without any changes in kidney or liver function markers [23]. Our data on organ damage markers also showed that treatment with 1 g resveratrol caused no changes to liver function markers (SGOT, SGPT, GGT, and ALP), as well as a kidney function marker (creatinine) in T2DM patients, indicating that short term resveratrol supplementation at a moderate to high dose had no adverse effects on liver and kidney. However, our study was only for 45 days, and longer studies would be necessary to confirm the safety of resveratrol at different doses. As speculated earlier, use of a lower dose (<1 g) after the 45-day treatment with 1 g resveratrol may eliminate or minimize, if any, resveratrol related toxicity in the long term. On the other hand, it should be mentioned that the control group which was on standard diabetic treatment alone had an increase in alkaline phosphatase (ALP) levels, when compared to the baseline values.

Glycated hemoglobin (HbA1c) levels in blood are another stable marker for blood glucose levels [36]. It must be noted that a decrease by one point in HbA1c levels observed in the intervention group is clinically very significant, given that this was achieved with 45 days of treatment. This result is comparable to decrease in HbA1c levels in T2DM patients with metformin (a frontline glycemic control drug) administration [37]. Accordingly, this data provides further evidence for the effectiveness of resveratrol supplementation in maintaining the lowered blood glucose levels during the study period. There was also a striking decrease in insulin levels, along with significant reductions in HOMA-IR and HOMA-*β* in the intervention group, suggesting that addition of resveratrol to standard antidiabetic therapy is beneficial in lowering insulin levels and improving insulin sensitivity and beta cell function in T2DM patients. Brasnyó et al. [22] did not observe any difference in serum insulin values with resveratrol treatment in type 2 diabetic patients, but they noted a significant reduction in HOMA-IR values; a lack of effect of resveratrol on serum insulin values may be attributed to the very low dose of resveratrol (10 mg/day) used in their study. Although HOMA can be used in subjects treated with insulin or in subjects on insulin secretagogues for determining *β*-cell function over time [38], our results need to be interpreted with caution. 

Type 2 diabetes is associated with increased risk of ischemic heart disease, and higher (than normal) LDL cholesterol levels and lower HDL levels, are independent risk factors for ischemic heart disease [39, 40]. Our data shows that HDL levels were significantly increased in the intervention group. The increase in HDL levels by 4.75 mg/dL with resveratrol treatments was comparable to that achieved with nicotinic acid [41]. There were trends towards a decrease, but no significant changes were observed in triglycerides, cholesterol, and LDL levels with resveratrol supplementation, while a significant increase in LDL levels was observed in the control group. Metformin, glibenclamide, and insulin are all known to have beneficial or no effect on the lipid profiles [42]; therefore, other independent factors may have contributed to the observed increase in the control group. 

Subgroup analyses showed that resveratrol may have an added effect when used in combination with glibenclamide and metformin. However, the power of the current study for subgroup analyses is weak; hence, the results should not be overinterpreted [43]. 

## 5. Conclusion and Summary

**Figure 2:**
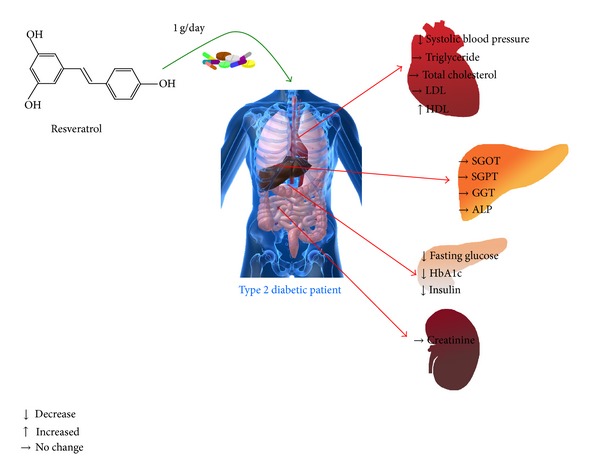
Summary of the effect of resveratrol supplementation in type 2 diabetic patients for 45 days.

The results of this study clearly demonstrate that resveratrol supplementation in the presence of standard antidiabetic medication has major benefits in T2DM patients, which include a pronounced lowering of blood glucose, HbA1c, insulin levels, and insulin resistance, as well as improvement in HDL levels (Figure 2). Unlike earlier reports [21–23] which showed mild effects on hyperglycemia and hyperinsulinemia, our study highlights the marked changes and the potential of resveratrol as an antidiabetic molecule. It is also important to note the beneficial effects of resveratrol observed on metabolic parameters, despite the fact that there was no appreciable effect on body weight or body composition. Some of the observed reductions in HbA1c and HDL with resveratrol supplementation are very significant that they can be compared to benefits achieved with front line antidiabetic drugs. Other important observations which stem from this study are that: (a) 1 g/day of resveratrol supplementation for 45 days had no adverse effects in type 2 diabetic patients and (b) resveratrol not only complemented standard antidiabetic medication but also provided added protection (over standard antidiabetic therapy).

### 5.1. Study Limitations

This is a pilot study which examined the antidiabetic effects of resveratrol over the short term, and only one dose of resveratrol was tested. There was no followup to check the metabolic parameters of the patients after the resveratrol wash-out period. The study lacks the investigation of cellular mechanisms underlying the antidiabetic effects of resveratrol. Extensive toxicological screening was not done on subjects to confirm the safety of resveratrol administration, and the safety of administering 1 g dose of resveratrol over the long term has not been established. 

In summary, the present study supports the strong antidiabetic effect of resveratrol reported in numerous animal studies, as well as the effects observed in the human studies. It also supports the case for resveratrol supplementation over a short term. Nevertheless, well-designed clinical trials with resveratrol supplementation in a larger T2DM population and over a longer duration are required to recommend the use of resveratrol independently or as an adjunct in diabetic population. 

## Figures and Tables

**Table 1:** Baseline clinical characteristics of the intervention and control groups before resveratrol or placebo supplementation.

	Control group	Intervention group	*P* value
Age (yrs)	51.81 ± 6.99	52.45 ± 6.18	0.697
Sex (female/male)	16/17	17/16	0.806
Duration of disease (yrs)	5.39 ± 1.36	5.81 ± 1.53	0.239
Family history of diabetes (yrs)	24 (77.4%)	15 (50.0%)	0.024*
Smoking (*n*)	4 (12.9%)	7 (21.90%)	0.348
History of hypertension (*n*)	8 (27.6%)	9 (29.0%)	0.901

Data is presented as means ± SD. For history of diabetes, smoking, and hypertension, the bracketed value corresponds to the total percentage of participants in respective groups. *P < 0.05 versus control group.

**Table 2:** Anthropometric, clinical, and biochemical parameters for placebo and resveratrol groups before and after resveratrol supplementation.

	Control/placebo group			Intervention/resveratrol group		
	Baseline	After 45 days	*P* value	Baseline treatment	After treatment	*P* value
Body weight (kg)	76.60 ± 14.27	76.60 ± 14.16	0.809	74.26 ± 11.39	74.48 ± 11.34	0.712
BMI (kg/m^2^)	27.83 ± 4.21	27.69 ± 4.15	0.332	27.05 ± 3.13	27.16 ± 3.13	0.395
Systolic blood pressure (mmHg)	129.31 ± 15.16	130.68 ± 13.21	0.147	129.03 ± 14.91	121.45 ± 10.26	<0.0001*
Diastolic blood pressure (mmHg)	78.58 ± 15.39	81.55 ± 5.84	0.279	76.93 ± 19.54	78.54 ± 6.35	0.169
Fasting glucose (mg/dL)	151.24 ± 51.52	161.13 ± 53.16	0.002*	175.74 ± 49.63	140.80 ± 39.74	<0.0001*
Insulin (*µ*IU/mL)	9.04 ± 5.35	8.77 ± 4.16	0.642	10.20 ± 4.33	5.37 ± 2.62	<0.0001*
HbA1c	8.30 ± 1.43	8.50 ± 2.46	0.764	8.6 ± 1.390	7.60 ± 1.32	<0.0001*
HOMA-IR	3.20 ± 2.37	3.43 ± 1.83	0.423	4.61 ± 2.77	1.91 ± 1.17	<0.0001*
HOMA-*β*	36.13 ± 8.45	35.68 ± 7.95	0.039	32.15 ± 5.32	25.80 ± 4.43	0.009*
Triglyceride (mg/dL)	134.69 ± 45.61	123.13 ± 43.27	0.145	160.1 ± 58.96	142.28 ± 52.61	0.051
Total cholesterol (mg/dL)	168 ± 41.97	175.34 ± 41.31	0.424	203.61 ± 52.70	192.28 ± 53.13	0.156
HDL-cholesterol (mg/dL)	41.73 ± 9.52	39.69 ± 10.83	0.133	41.40 ± 8.35	46.15 ± 8.40	0.001*
LDL-cholesterol (mg/dL)	107.95 ± 31.67	117.18 ± 29.88	0.003*	134.04 ± 36.18	122.71 ± 38.19	0.106
SGOT (IU/L)	24.0 ± 5.47	25.0 ± 6.71	0.212	26.0 ± 5.87	26.0 ± 7.56	0.837
SGPT (IU/L)	19.44 ± 8.79	21.65 ± 8.67	0.202	21.45 ± 7.91	22.61 ± 9.74	0.365
GGT (IU/L)	30.82 ± 17.79	29.93 ± 17.01	0.545	32.12 ± 15.32	33.38 ± 17.92	0.441
ALP (IU/L)	169.37 ± 52.63	189.41 ± 48.38	0.001*	185.29 ± 59.35	190.64 ± 47.55	0.372
Creatinine (mg/dL)	0.92 ± 0.24	0.97 ± 0.25	0.281	0.96 ± 0.24	0.90 ± 0.21	0.098

Data is presented as means ± SD. *P < 0.05 versus before treatment. HOMA-*β*: homeostasis model of assessment for beta cell function; HOMA-IR: homeostasis model of assessment for insulin resistance; HbA1c: Hemoglobin A1c; HDL: high density lipoprotein; LDL: low density lipoprotein; TG: triglyceride; SGOT: serum glutamate oxaloacetate transaminase; SGPT: serum glutamate pyruvate transaminase; GGT: gamma-glutamyltransferase; ALP: alkaline phosphatase.

**Table 3:** Comparison of changes in the anthropometric, clinical, and biochemical parameters during the study period between the intervention and control groups.

	Control group	Intervention group	*P* value
Body weight	0.02 ± 0.64	0.21 ± 0.61	0.220
BMI	−0.01 ± 0.89	0.12 ± 0.43	0.092
Systolic blood pressure	1.37 ± 4.98	−7.58 ± 8.04	<0.0001*
Diastolic blood pressure	2.96 ± 14.48	1.61 ± 6.37	0.638
Fasting glucose	9.89 ± 15.72	−34.93 ± 29.53	<0.0001*
Insulin	−0.27 ± 3.15	−4.82 ± 4.83	<0.0001*
HbA1c	0.01 ± 0.67	−1.20 ± 1.56	<0.0001*
HOMA-IR	0.22 ± 1.50	−2.69 ± 2.79	<0.0001*
HOMA-*β*	−2.74 ± 2.84	−9.16 ± 12.27	0.549
Triglyceride	−11.56 ± 36.71	−17.81 ± 39.87	0.591
Total cholesterol	6.69 ± 40.68	−11.33 ± 35.24	0.121
HDL-cholesterol	−2.4 ± 6.26	4.75 ± 5.83	0.001*
LDL-cholesterol	9.22 ± 12.88	−11.33 ± 30.65	0.006*
SGOT	−2.34 ± 9.96	0.83 ± 7.90	0.174
SGPT	2.20 ± 9.09	1.16 ± 7.03	0.619
GGT	−0.89 ± 7.88	1.25 ± 8.97	0.329
ALP	20.03 ± 28.15	5.35 ± 32.86	0.069
Creatinine	0.05 ± 0.28	−0.06 ± 0.21	0.062

Data is presented as means ± SD. *P < 0.05 versus control group. HOMA-*β*: homeostasis model of assessment for beta cell function; HbA1c: Hemoglobin A1c; HDL: high density lipoprotein; LDL: low density lipoprotein; TG: triglyceride; SGOT: serum glutamate oxaloacetate transaminase; SGPT: serum glutamate pyruvate transaminase; GGT: gamma-glutamyltransferase; ALP: alkaline phosphatase.

**Table 4:** Comparison of changes in the clinical and biochemical parameters during the study period between the intervention and control groups across glibenclamide, metformin, and metformin + glibenclamide treatment subgroups.

	Glibenclamide			Metformin			Glibenclamide + metformin		
	Control group	Intervention group	*P* value	Control group	Intervention group	*P* value	Control group	Intervention group	*P* value
Systolic blood pressure	0.01 (−5.00, 1.25)	−10.00 (−16.25, −3.75)	0.016*	0.01 (−1.25, 10.00)	−10.00 (−10.00, 0.01)	0.043*	0.01 (0.01, 5.00)	−10.00 (−12.50, −2.50)	0.003*
Fasting glucose	13.0 (4.50, 31.50)	−25.00 (−84.50, −5.75)	<0.0001*	2.00 (−3.50, 12.00)	−25.00 (−32.00, −10.00)	0.005*	7.00 (1.00, 22.00)	−32.00 (−47.50, −22.50)	<0.0001*
Insulin	0.06 (−3.85, 5.39)	−4.42 (−7.85, −2.31)	0.042*	0.73 (0.06, 1.05)	−1.32 (−3.39, −0.04)	0.088	0.37 (0.32, 0.95)	−7.71 (−9.31, −1.96)	0.002*
HbA1c	−0.15 (−1.04, 0.200)	−1.55 (−2.22, −0.97)	0.031*	−0.15 (−0.70, 0.22)	−0.60 (−2.00, −0.02)	0.133	0.05 (−0.10, 0.50)	−1.10 (−2.05, −0.90)	<0.0001*
HOMA-IR	0.17 (−1.53, 3.69)	−2.12 (−5.07, −1.16)	0.022*	0.27 (0.15, 0.65)	−0.86 (−1.25, −0.31)	0.043*	0.24 (−0.26, 0.56)	−2.86 (−5.22, −1.12)	<0.0001*
HDL-cholesterol	−1.65 (−5.77, 1.40)	6.25 (2.82, 11.26)	0.003*	0.10 (−4.07, 2.95)	4.60 (1.40, 12.05)	0.043*	−2.80 (−4.70, 0.60)	6.10 (0.35, 11.20)	<0.0001*
LDL-cholesterol	8.0 (5.50, 18.0)	−17.50 (−35.75, −10.50)	0.005*	4.50 (−1.25, 20.75)	−7.0 (−70.00, 2.00)	0.007*	1.00 (−2.00, 4.00)	−9.00 (−32.00, 1.50)	0.030*

Data is presented as medians (Interquartile ranges). *P < 0.05 versus control group. HOMA-IR: homeostasis model of assessment for insulin resistance; HbA1c: Hemoglobin A1c; HDL: high density lipoprotein; LDL: low density lipoprotein.
